# Supplementary material for: Analysis of Policies to Protect the Health of Urban Refugees and Asylum Seekers in Thailand: A Qualitative Study and Delphi Survey
Source: Int J Environ Res Public Health. 2021 Oct 9;18(20):10566. doi: 10.3390/ijerph182010566 (PMC8535300; doi:10.3390/ijerph182010566)
Supplement: Supplementary file 1 [file ijerph-18-10566-s001.zip › Sataporn_Supplementary file 1_210707.pdf]

## Supplementary file 1: questionnaires for Delphi survey

| Domain | Question detail | Opinion (1 least agree ←→5 most agree) | | | | | Additional comments |
|---|---|---|---|---|---|---|---|
| | | 1 | 2 | 3 | 4 | 5 | |
| Health financing | 1. Urban refugees should have rights to receive health services provided by the public facilities and the Ministry of Public Health should be the main authority responsible for any expenses incurred. | | | | | | |
| | 2. Urban refugees should have rights to receive health services provided by the public facilities and the National Health Security Office should be the main authority responsible for any expenses incurred. | | | | | | |
| | 3. Urban refugees should have rights to receive health services provided by the public facilities; however, any expenses incurred should be mainly borne by the clients themselves. | | | | | | |
| | 4. Urban refugees should have rights to receive health services provided by the public facilities. Any expenses incurred should be borne by the UNHCR. | | | | | | |
| | 5. Urban refugees should have rights to receive health services provided by the public facilities. Any expenses incurred should be borne by the NGOs. | | | | | | |
| Benefit package | 6. The benefit package for urban refugees should cover only care for basic diseases, for instance, general outpatient care and essential vaccination | | | | | | |
| | 7. The benefit package for urban refugees should cover only treatment for emergency conditions and accidents. | | | | | | |
| | 8. The benefit package for urban refugees should cover only treatment for communicable diseases with public health threat, such as measles, tuberculosis, and influenza. | | | | | | |
| | 9. The benefit package for urban refugees should be comprehensive and covering all types of care (from general outpatient and inpatient treatments to high-cost care), similar to the benefits for Thai citizens. | | | | | | |
| | 10. The benefit package for urban refugees should be cover as same as benefit package for migrant workers. | | | | | | |
| | 11. The benefit package for urban refugees should be cover treatment for mental diseases or other mental problems, such as stress and mental disorder. | | | | | | |
| Insurance | 12. Urban refugees should have rights to purchase the insurance for migrant workers of the Ministry of Public Health. | | | | | | |

| Domain | Question detail | Opinion (1 least agree ←→5 most agree) | | | | | Additional comments |
|---|---|---|---|---|---|---|---|
| | | 1 | 2 | 3 | 4 | 5 | |
| | 13. Urban refugees should have rights to be enrolled in the Social Security Scheme which is managed by the Ministry of Labour. | | | | | | |
| | 14. Urban refugees should have rights to be enrolled in the Universal Coverage Scheme which is managed by the National Health Security Office. | | | | | | |
| | 15. Urban refugees should have rights to buy health insurance from private organisations | | | | | | |
| Other aspects of well-being | 16. Children of urban refugees should have rights to enjoy basic education at the same level as Thai children | | | | | | |
| | 17. Urban refugees should have rights to get work permit and be able to work in Thailand children. | | | | | | |
| | 18. Urban refugees should have their name and family profile recorded in the civil registry similarly to Thai citizens. | | | | | | |
| | 19. Urban refugees should have rights to live outside detention center or refugee center. | | | | | | |
| position of Thai government | 20. Thailand should ratify the United Nations 1951 UNHCR Refugee Convention. | | | | | | |
| | 21. The current position of Thai government on urban refugees is appropriate. | | | | | | |
