# Supplementary figures and images for: Analysis of Policies to Protect the Health of Urban Refugees and Asylum Seekers in Thailand: A Qualitative Study and Delphi Survey
Source: Int J Environ Res Public Health. 2021 Oct 9;18(20):10566. doi: 10.3390/ijerph182010566 (PMC8535300; doi:10.3390/ijerph182010566)

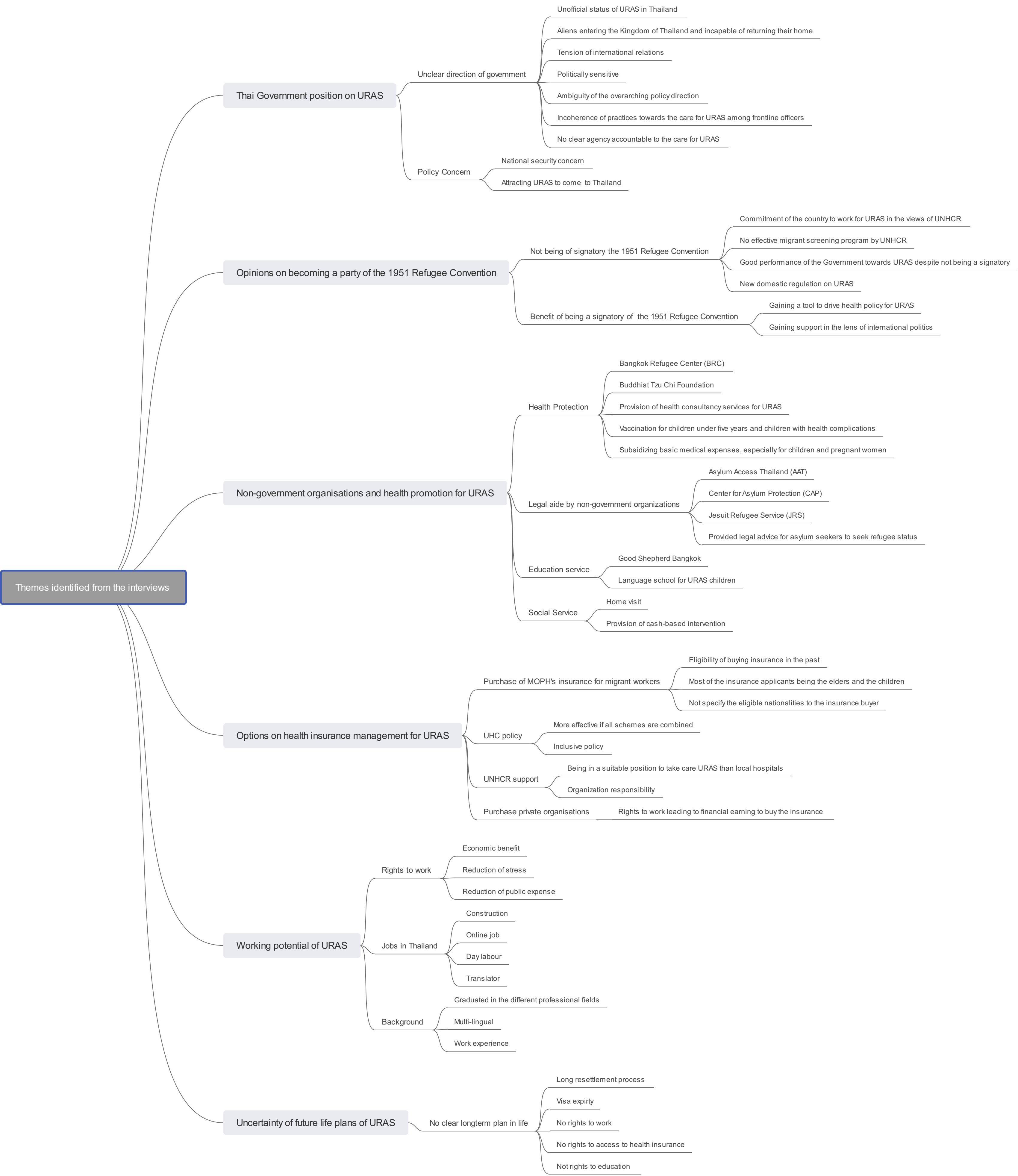

Supplement: Supplementary file 1 [file ijerph-18-10566-s001.zip › Sataporn_Supplementary file 2.pdf]
